# Complete Genome Sequence of O/VN1/2014, a Foot-and-Mouth Disease Virus of Serotype O Isolated in Vietnam in 2014

**DOI:** 10.1128/MRA.01343-18

**Published:** 2019-02-07

**Authors:** HyunJi Lee, JinJu Nah, Soyoon Ryoo, Taeseong Kim, Sumee Lee, Semin Jung, Jinwoo Lee, Hye-jin Park, Byeong-suk Ha, Jeong-won Lee, Tho Dang Nguyen, Long Thanh To, Sung-Hwan Wee, Bok Kyung Ku

**Affiliations:** a Foot-and-Mouth Disease Division, Animal and Plant Quarantine Agency, Gimcheon-si, Gyeongsangbuk-do, Republic of Korea; b Department of Animal Health, National Center for Veterinary Diagnosis, Hanoi, Vietnam; Loyola University Chicago

## Abstract

In this article, we report the complete genome sequence of foot-and-mouth disease virus (FMDV) strain O/VN1/2014 isolated in Vietnam (Lao Cai) in 2014. The virus belongs to serotype O, topotype South East Asia (SEA), and genotype Mya-98 (O/SEA/Mya-98). It is the latest complete genome information for the genotype O/SEA/Mya-98 in Vietnam since 2009.

## Abstract (precis)

In this article, we report the complete genome sequence of foot-and-mouth disease virus (FMDV) strain O/VN1/2014 isolated in Vietnam (Lao Cai) in 2014. The virus belongs to serotype O, topotype South East Asia (SEA), and genotype Mya-98 (O/SEA/Mya-98).

## ANNOUNCEMENT

Foot-and-mouth disease viruses (FMDVs) are highly contagious in cloven-hoofed animals, such as cattle and pig, and are classified under seven serotypes (O, A, Asia1, C, SAT1, SAT2, and SAT2). FMDVs are prevalent in most Southeast Asian countries, including Vietnam (1, 2). Serotype O and A of FMDV have most commonly been occurring endemically, and the O/SEA/Mya-98 genotype was one of the indigenous genotypes and has shown a high incidence rate in Vietnam (3, 4).

In this study, we isolated the FMDV strain O/VN1/2014 from an epithelial sample of a pig with clinical symptoms of FMDV in northern Vietnam (Lao Cai Province) in October 2014. The virus was cultured until passage 2 in the cell line ZZR-127, originated from a fetal goat tongue epithelium (5). Viral RNA was extracted with an RNeasy mini kit (Qiagen), and the full genome was amplified by PCR using a Qiagen one-step reverse transcriptase PCR (RT-PCR) kit and 19 overlapping pairs of FMDV-specific primers, based on the sequence of O/SKR/JC/2014 (GenBank accession no. MG257782). The PCR products were subjected to direct sequencing using a ABI 3730XL instrument with a BigDye Terminator v3.1 cycle sequencing kit. The sequences of the PCR products were assembled by Clustal W multiple alignment with default parameters using the software BioEdit (version 7.2.5.). A single open reading frame (ORF; protein-coding region) was predicted by comparing the sequence of O/SKR/JC/2014 with the FMDV reference sequence (GenBank accession no. MG257782). The sequence homology was determined by BLAST via the NCBI website.

The complete genome of O/VN1/2014 was 8,131 nucleotides (nt) in length, including a 5′ untranslated region (UTR) of 1,011 nt with a poly(C) of 19 nt and a 3′ UTR of 121 nt with a poly(A) tail of 26 nt. A single ORF of 6,999 nt was predicted to encode a polyprotein of 2,332 amino acids (aa) containing of four structural (VP4, VP2, VP3, and VP1) and eight nonstructural proteins (L, 2A, 2B, 2 C, 3A, 3B, 3C, and 3D). O/VN1/2014 belongs to the topotype SEA and genotype Mya-98 of FMDV serotype O (O/SEA/Mya-98). The virus is most closely related to a Korean FMDV isolate, O/SKR/JC/2014 in the public database, with a 97.3% nt identity and no indels at the ORF (6,810/6,999 nt). But, it is less related to a Vietnam FMDV isolate, O/VN/QB88/2009 (GenBank accession no. GU582115; only with ORF), with a 90.1% nt identity at the ORF (6,306/6,999 nt).

Our findings confirmed that at least two genetically different O/SEA/Mya-98 genotypes, one closely related to the Korean FMDV strain from 2014, have been circulating in Vietnam. The complete genome sequence of O/VN1/2014 could provide the valuable information for further study of the genetic diversity and the antigenic relationship of FMDVs in the Asian region, with the ultimate aim to control and prevent FMDV.

### Data availability.

The complete genomic sequence of O/VN1/2014 has been deposited in GenBank under the accession no. MH845413.

## References

[1] World Organisation for Animal Health, FAO. 2012 The global foot and mouth disease control strategy. Strengthening animal health system through improved control of major diseases, p. 43. FAO, Rome, Italy.

[2] Le VP, Nguyen T, Lee K-N, Ko Y-J, Lee H-S, Nguyen VC, Mai TD, Do TH, Kim S-M, Cho I-S, Park J-H 2010 Molecular characterization of serotype A foot-and-mouth disease viruses circulating in Vietnam in 2009. Vet Microbiol 144:58–66. doi:10.1016/j.vetmic.2009.12.033. 20097490

[3] Lee KN, Nguyen T, Kim SM, Park JH, Do HT, Ngo HT, Mai DT, Lee SY, Nguyen CV, Yoon SH, Kweon CH, Cho IS, Kim H 2011 Direct typing and molecular evolutionary analysis of field samples of foot-and-mouth disease virus collected in Viet Nam between 2006 and 2007. Vet Microbiol 147:244–252. doi:10.1016/j.vetmic.2010.06.030. 20667669

[4] Le VP, Vu TTH, Duong HQ, Than VT, Song DS 2016 Evolutionary phylodynamics of foot-and-mouth disease virus serotypes O and A circulating in Vietnam. BMC Vet Res 12:269. doi:10.1186/s12917-016-0896-0. 27894299 PMC5126991

[5] Brehm KE, Ferris NP, Lenk M, Riebe R, Haas B 2009 Highly sensitive fetal goat tongue cell line for detection and isolation of foot-and-mouth disease virus. J Clin Microbiol 47:3156–3160. doi:10.1128/JCM.00510-09. 19656987 PMC2756941

